# Eosinophilic Fasciitis: What Matters in Management in a Developing Country—A Case Report with Two and a Half-year Follow-up

**DOI:** 10.3329/jhpn.v30i1.11292

**Published:** 2012-03

**Authors:** Md. Nazrul Islam, Md. Ariful Islam, Syed Jamil Abdal, Mohammad Abul Kalam Azad, Abul Khair Ahmedullah, Syed Atiqul Haq

**Affiliations:** Department of Medicine, Bangabandhu Sheikh Mujib Medical University, Shahbagh, Dhaka 1000, Bangladesh

**Keywords:** Diabetes, Eosinophilia, Eosinophilic fasciitis, Bangladesh

## Abstract

Eosinophilic fasciitis is an uncommon disorder of unknown aetiology and poorly-understood pathogenesis. Since 1974, over 250 cases of eosinophilic fasciitis have been reported worldwide. The first case of eosinophilic fasciitis from Bangladesh is reported here. The challenges of diagnosis, treatment, and follow-up, including family and social support, are discussed.

## INTRODUCTION

Eosinophilic fasciitis is a rare, localized fibrosing disorder of the fascia of unclear aetiology and pathophysiology (1). It is associated with peripheral eosinophilia, hypergammaglobulinaemia, elevated erythrocyte sedimentation rate, erythema, swelling, induration of the extremities, and no visceral changes (2). In 1974, Shulman provided an early description of eosinophilic fasciitis as a disorder characterized by peripheral eosinophilia and fasciitis that could be differentiated from scleroderma by the distinctive pattern of skin involvement that spares the digits, involves the fascia rather than dermis, and is not accompanied with the Raynaud phenomenon (3). Biopsy showed a conspicuous thickening of the fascia between the subcutis and the muscle, with an intense infiltration of lymphocytes and plasma cells and a good response to corticosteroid therapy, suggesting that this was a distinct rheumatic disease syndrome. Based on the striking histological changes in the subcutaneous fascia, Rodnan proposed that this condition be termed ‘eosinophilic fasciitis’ (1). Since 1974, over 250 patients with eosinophilic fasciitis have been reported worldwide (4).

## CASE REPORT

A 40-year-old non-hypertensive mother (housewife) of three children was admitted to the Bangabandhu Sheikh Mujib Medical University, a tertiary-care hospital in Dhaka, in 2008. About a year prior to hospitalization, the patient first noticed itchy reddish localized swelling in the medial aspect of the right forearm. She described her symptoms as a feeling of an insect moving within it and felt some comfort when hot compression was used. Four months after the onset of the illness, she noticed blackish pigmentation at the margin of the lesion with profound itching. The lesion progressed and involved the wrist to the lower part of the right arm. The patient also noticed numbness in the whole right arm with pain and tightness. Due to lack of improvement, her general physician pres-cribed betamethasone tablet (0.5 mg) three times daily. With betamethasone, the patient noticed remarkable improvement in terms of reduction in pain and pigmentation over four months. However, she developed generalized swelling of the body, for which she was hospitalized for further evaluation.

On admission, other than swelling of the whole body, she complained of generalized aches and weakness. There were no symptoms to suggest pulmonary, renal, cardiac or gastrointestinal involvement. Her past medical history was unremarkable, and none of her family members was suffering from any disease of connective tissues. She came from a lower middle-class family and lived in a second class government staff quarter. She did not give any history of smoking, tobacco-use, or intake of alcohol. On examination, the pulse rate was 96 beats per minute; blood pressure was 115/75 mm Hg; and she had a puffy face and mild pedal oedema. Diffuse blackish pigmentation was seen over the anterior aspect of her both upper limbs, more on right arm, palmar creases, and knuckle of the fingers. Muscle power was 4/5 in proximal muscles of all limbs. There was moderate tenderness in the second, third, fourth, and fifth proximal and distal interphalangeal and metacarpal joints. Her forearms were tight, grooving over the attachment of fascia, more marked on the right side. From her presentation, possibilities of eosinophilic fasciitis, scleroderma, and the eosinophilia-myalgia syndrome were suspected. Examination of her complete blood count showed a marked eosinophilia (Table). Her blood sugar, liver and kidney function tests were normal. A skin biopsy was reported as follows: “atrophic epidermis; dermis reveals thick bundles of collagen with atrophied adnexa. The papillary dermis shows infiltration of chronic inflammatory cells and melanophages. Infiltration of eosinophil, lymphocytes, polymorph, and mast cells was noted in the subcutaneous tissue. The features are compatible with scleroderma”. Subsequently, a muscle biopsy was done. Its microscopic appearance showed skeletal muscle and fascia. The fascia was thick due to oedema and fibrosis. There was a moderate infiltrate of lymphocytes, eosinophil mixed with a small number of histiocytes, and plasma cells. The features were compati-ble with eosinophilic fasciitis. In both the biopsies, direct immunofluorescence showed no deposition of immunoglobulins (IgM, IgA, IgG), C3, or fibrino-gen. Serological markers, such as serum creatine kinase (CK), anti-Scl 70, anti-centromere antibody, rheumatoid factor, and antinuclear antibody, were negative. The serum immunoglobulin levels were within the normal range. Her ECG, PA view of chest x-ray, AP and lateral view x-ray of lumbar spines, and ultrasonography of the whole abdomen were normal. Initially, her serum alanine transaminase was 64 U/L, which later became 15 U/L, and serum creatinine was 1 mg/dL.

**Table.** Total and differential counts of WBC and blood glucose at different visits

Test	10.08.08	26.09.08	12.11.08	15.01.09	21.04.09	28.08.09	27.05.10	11.09.10	08.01.11
ESR: mm in 1st hour	28	25	-	25	40	15	45	15	25
TC WBC (x 10^9^/L)	17.1	10	10	8	9.5	10	9	9.5	9.3
DC WBC (%) Eosinophil	36	50	08	02	12	04	10	07	18
FBS (mmol/L)	-	-	-	-	-	-	-	7.8	7.0
2 AFB (mmol/L)	6	-	5.5	-	-	6.8	-	14.2	10

AFB=After breakfast;

DC=Differential count;

ESR=Erythrocyte sedimentation rate;

FBS=Fasting blood sugar;

TC=Total count;

WBC=White blood cell

She was discharged with oral prednisolone 20 mg daily in the morning, hydroxychloroquine 200 mg twice daily, calcium carbonate 500 mg twice daily, and vitamin D3 200 IU twice daily, with a follow-up schedule at an interval of 4-6 weeks for three months and then three-monthly.

The response to treatment was remarkable. The eosinophil count came down to normal with relief of pain and improvement in the tightening of the skin. Noting improvement, steroids were reduced by 2.5 mg per week. An eosinophil count of less than 10% was achieved with 10 mg prednisolone daily. Further reduction to 7.5 mg failed to maintain the eosinophil count at 10%. So, she was kept on 10 mg prednisolone daily as a maintenance dose. Subsequently, the patient was irregular in follow-up.

After about two years, she noticed gain in weight and nocturnal polyuria. On evaluation, her fasting blood glucose was 7.8 mmol (Table), and she was diagnosed as a case of type II diabetes mellitus, for which oral anti-diabetic agents with lifestyle modification, including diet and exercise, were prescribed. Ultimately, 10 mg prednisolone was enough to maintain the eosinophil count near normal but good glycaemic control remained a problem with oral anti-diabetic agents. She continued taking hydroxychloroquine at a dose of 200 mg b.i.d with no ocular problem/toxicity.

In subsequent follow-up, she described family disharmony and developed hypertension. At that stage, she became more irregular in routine follow-up and refused to take any additional medicine because of financial constraints, lack of family support, and profound depression.

## DISCUSSION

Eosinophilic fasciitis is a scleroderma-like syndrome of unknown cause characterized by inflammation, followed by sclerosis of the dermis, subcutis, and deep fascia. The disease affects adults. Patients usually have an abrupt onset of symmetrical tenderness and swelling of the extremities, rapidly followed by induration of the skin and subcutaneous tissue without Raynaud's phenomenon. Carpal tunnel syndrome and low-grade myositis with normal creatine kinase and flexion contractures may develop later. A marked eosinophilia is found in the early stage of the disease and subsequently decreases. A full thickness biopsy, consisting of skin, fascia, and superficial muscle, shows perivascular infiltration of histiocytes, eosinophils, lymphocytes, and plasma cells. The spontaneous improvement and occasionally complete remission might occur after 2-5 years of disease. Some patients suffer from persistent disease while others are left with flexion contractures (5). The majority of patients with eosinophilic fasciitis have peripheral blood eosinophilia during the acute phase of disease. In one series, 33 of 52 patients had eosinophilia (6) and elevated erythrocyte sedimentation rate (29%), and polyclonal hypergammaglobulinaemia (35%) can also be found (7). Antinuclear antibody positivity has not been reported previously in eosinophilic fasciitis with any consistency, and rheumatoid factor is almost always negative. Definitive diagnosis requires histopathological examination from a full-thickness (epidermis to muscle) biopsy (8).

The presentation of this patient with itchy reddish and swollen skin lesion was not typical of eosinophilic fasciitis at the beginning, leading to a reasonable delay in diagnosis, especially as it is a rare disease. During the course of her illness, evolving features, especially peripheral eosinophilia, provoked the possibility of a diagnosis of eosinophilic fasciitis. The course of her illness was progressive, resulting in marked tightening of the skin and subsequent impairment of joint mobility. An evident groove sign in skin and absence of Raynaud's phenomenon were striking. The disease spared her face, hands, and feet. Her antinuclear antibody and rheumatoid factor test was negative. Full-thickness biopsy (epidermis to muscle) was consistent with diagnosis.

The spontaneous remission rate in patients with eosinophilic fasciitis is 10-20% from the time of presentation or relapse after discontinuing corticosteroid therapy (9). In one series, haematological disorders other than eosinophilia were present in 5 of 52 patients (7). Haematological abnormalities that have been described, in association with eosinophilic fasciitis, include aplastic anaemia, acquired amegakaryocytic thrombocytopaenia, myeloproliferative disorders, myelodysplastic syndromes, lymphoma, leukaemia, and multiple myeloma (7). However, there was no haematological abnormality in our patient. There is substantial agreement among published cases and case series that corticosteroids are the first-line treatment for eosinophilic fasciitis and are usually effective in over 70% of cases. Other treatments include non-steroidal anti-inflammatory drugs, D-penicillamine, chloroquine, cimetidine, methotrexate, azathioprine, cyclosporine A, infliximab, UVA-1, and bath PUVA (10,11).

Lakhanpal *et al.* reported 52 cases of Raynaud's phenomenon, and only 59% of them had a satisfactory response to prednisolone alone (40-60 mg/day in divided daily doses) (12). Abnormalities found through laboratory tests, including elevated erythrocyte sedimentation rate, peripheral eosinophilia, and hypergammaglobulinaemia, reverted to normal with prednisolone therapy in most patients (12). However, corticosteroid resistance has been encountered in the setting of paraneoplastic and in-graft versus host disease (13).

Response to steroid and hydroxychloroquine was good in our case but a low dose of prednisolone (10 mg) was needed to maintain an eosinophil count of less than 10%. The patient developed diabetes and hypertension after about two years of taking steroid. While taking steroid, she needed glimepi-ride 4 mg per day to manage diabetes. She became partly refractory to ongoing treatment and developed two more co-morbid illnesses most likely due to the steroid. At the end, her long suffering and co-morbidities resulted in family disharmony, and she became profoundly depressed. She refused further medication and was lost to follow-up.

Eosinophilic fasciitis can present with various symptoms. A strong suspicion and a proper approach help in its early diagnosis. In a developing country like Bangladesh, rheumatology gets little space in the graduate curriculum, and a graduate gets little or no exposure to most common rheumatic illnesses during his/her studies. Chronic and rare rheumatic illnesses need a strong index of suspicion that can only be achieved through training, refresher courses, and easy access to medical journals. In Bangladesh, there is no training programme to update our graduate practitioners, and there is no central system of accessing medical publications by our health practitioners. The injudicious use of steroids is an issue among our physicians due to the lack of knowledge and awareness, and this blurred the clinical presentation of the disease in our case, leading to a further delay in accurate diagnosis. Patients often continue taking steroids without following the instructions of their physicians, i.e. they do it by self-prescribing, due to their comfort. Besides, drugs are easily available from pharmacies.

Chronic illnesses are now a major health issue in both developed and developing countries. Many chronic illnesses are expensive to manage, and this is often the greatest barrier to their management (14). People who fall ill often face a dire choice: either to suffer death without treatment or to seek treatment and push their family into poverty (15). This patient could have been better managed by agents, such as azathioprine, methotrexate, and cyclosporine, with no impact on blood sugar; however, all are costly (azathioprine and cyclosporine) due to price and follow-up laboratory tests (metho-trexate and cyclosporine). In developing countries like Bangladesh, households often sell their possessions to cover healthcare costs, particularly when chronic diseases require long-term costly treatment (16). Supporting family members who live with chronic illness requires recognition of the complexity of the illness experience and the nature of social support (17).

### Conclusion

We recommend mass awareness and familial, social and government support to manage chronic rheumatic diseases reported in this study.
